# Retraction Note: New insights into the microscopic interactions associated with the physical mechanism of action of highly diluted biologics

**DOI:** 10.1038/s41598-022-24707-3

**Published:** 2022-11-29

**Authors:** Kristina N. Woods

**Affiliations:** Lehrstuhl Für BioMolekulare Optik, Ludwig-Maximilians-Universität, 80538 Munich, Germany

Retraction of: *Scientific Reports* 10.1038/s41598-021-93326-1, published online 02 July 2021

The Editors have retracted this Article.

After publication, concerns were raised about the nature of the samples used in this study, in particular that the cytokine and antibodies are diluted beyond the point at which any active molecules are expected to be present. Post-publication peer review confirmed that some of the methods used in this study are not sensitive enough to provide interpretable results at these concentrations. This means that without further corroborative evidence, the data presented in the paper are not sufficient to attribute the differences in the signal to the sample preparation method. The Editors therefore no longer have confidence in the results reported in this Article.

Kristina N. Woods disagrees with this retraction.

